# Phytochemical Profiling and Biological Assessment of the Aerial Parts from Three Mediterranean *Alkanna* Species (*A. orientalis*, *A. tinctoria*, *A. kotschyana*) in the Boraginaceae Family

**DOI:** 10.3390/plants13020278

**Published:** 2024-01-17

**Authors:** Christos Ganos, Gökhan Zengin, Ioanna Chinou, Nektarios Aligiannis, Konstantia Graikou

**Affiliations:** 1 Laboratory of Pharmacognosy and Chemistry of Natural Products, Department of Pharmacy, National & Kapodistrian University of Athens, Zografou, 15771 Athens, Greece; chris50ganos@hotmail.com (C.G.); ichinou@pharm.uoa.gr (I.C.); aligiannis@pharm.uoa.gr (N.A.); 2 Laboratory of Physiology and Biochemistry, Department of Biology, Science Faculty, Selcuk University, 42130 Konya, Turkey; gokhanzengin@selcuk.edu.tr

**Keywords:** *Alkanna orientalis*, *A. tinctoria*, *A. kotschyana*, aerial parts, Boraginaceae, phenolic compounds, antioxidant activity, enzyme inhibition

## Abstract

This study focuses on the phytochemical analysis of the aerial parts of three *Alkanna* species: *A. orientalis* (L.) Boiss., *A. tinctoria* Tausch. and *A. kotschyana* A. DC. (Boraginaceae) growing wild in the Mediterranean basin, as mostly the roots of the genus have been widely researched. Their methanol extracts were subjected to qualitative LC-MS analyses, resulting in the annotation of 28 different secondary metabolites, with 27 originating from *A. orientalis*, 25 from *A. tinctoria* and 23 from *A. kotschyana*. The detected metabolites are categorized into three chemical types: organic acids (2), flavonoids and their glycosides (17), and caffeic acid derivatives (9). Furthermore, the chemical profiles of the three species are discussed chemotaxonomically. Caffeic acid and its derivatives, along with glucosides of quercetin and kaempferol, were identified in all three studied species. Additionally, their total phenolic and flavonoid contents were determined. The antioxidant capacity was evaluated through various chemical assays, as well as their in vitro enzyme inhibitory properties towards cholinesterases (AChE and BChE), α-amylase and α-glucosidase. The results showed that *A. tinctoria* exhibited the strongest antioxidant activity (211 mgTE/g extract in DPPH and 366 mgTE/g extract in ABTS), probably due to its high total phenolic (53.3 mgGAE/g extract) and flavonoid (20.8 mgRE/g extract) content, followed by *A. kotschyana*. These chemical and biological findings provide valuable insights for potential promising applications of the aerial parts of the species outside of the well-known uses of their roots.

## 1. Introduction

Boraginaceae is a botanical family widely distributed all over the world, mainly in Europe and in the Mediterranean region as well as in Asia, comprising approximately 130 plant genera and 2300 species. Several plant species in the family have been widely used for their pharmacological properties, mostly due to the high content of naphthoquinones in their roots, along with secondary metabolites such as flavonoids and phenolic acid derivatives [1].

The genus *Alkanna*, belonging to the family Boraginaceae, is represented by 17 species in South Europe extending from the Mediterranean to Iran. Several plants from this genus have been widely studied with regard to their extensive use in traditional medicine. In the framework of our studies on Boraginaceae plants [1], we report herein the phytochemical analyses, through qualitative Liquid Chromatography/Quadrupole Time-of-Flight Tandem Mass Spectrometry (LC/Q-TOF-MS), of three native plants, growing wild in the Mediterranean basin (Turkey): *Alkanna orientalis* (L.) Boiss., *A. tinctoria* Tausch and *A. kotschyana* A. DC. Furthermore, we report their biological properties, including the determination of total phenolic and flavonoid content, the antioxidant capacity evaluated through various chemical assays (DPPH^•^, ABTS^•+^, CUPRAC, FRAP, phosphomolybdenum and ferrous ion chelating test), and their in vitro enzyme inhibitory properties towards cholinesterases (AChE and BChE), α-amylase and α-glucosidase.

*Alkanna orientalis* (L.) Boiss (syn. *Anchusa orientalis* L.) is a perennial plant, growing in rocky places in South Greece [1,2], and it is used in Arabia for the treatment of the sore throat [2]. The volatiles of the fresh aerial parts’ oil have been examined by GC and GC-MS and among the main components are β-eudesmol (36.9%), α-eudesmol (16.3%) and γ-eudesmol (14.1%) [2]. Furthermore, flavonoids (identified as sarothrin [3], kaempferol-3-glucoside, kaempferol-3-rutinoside, quercetin-3-glucoside, quercetin-3-rutinoside, kaempferol-3,6-dimethyl ether and small amounts of its 7-glucoside [4], kaempferol-3,4′-dimethylether (ermanin), kaempferol-3,7-dimethylether (kumatakenin), 6-methoxyquercetin-3,3′-dimethylether (jaceidin), 6-methoxykaempferol-3-methyl ether, 6-methoxykaempferol-3,7-dimethylether (penduletin) and kaempferol-3-methylether (isokaempferide)) [5]) and pyrrolizidine alkaloids (PAs) of the retronecine type [6] have previously been recorded. Moreover, *Alkanna* species have been characterized as a source of γ-linolenic acid [7]. *A. orientalis* leaf and flower extract has inhibited the growth of *Staphylococcus aureus* in a bioassay-guided fractionation, which resulted in the isolation of the flavonoid sarothrin (5,7,4′-trihydroxy-3,6,8-trimethoxyflavone), which inhibited the growth of both *Mycobacterium smegmatis* (MIC 75 M) and *S. aureus* (MIC > 800 M), and possessed efflux pump inhibitory activity [3]. Dyeing of different fabrics has been carried out using a water extract from the roots of the *A. orientalis* plant and several studies on phenolic composition and cytotoxic activity of ethanol and water extracts showed that the roots of the plant can be used in the textile industry safely [5,8].

*Alkanna tinctoria* Tausch (syn. *Anchusa tuberculata* (Forssk.) Meikle) or alkanet or dyers’ bugloss/alkanet, is a perennial herbaceous plant distributed in Asia Minor, and in Southern Europe, especially in the Mediterranean region. Because of the production of alkannin pigment (a naphthoquinone), the roots of the plant have a long history as they have been widely used in the food industry for coloring confectionery and wines and as food additives, as well as in cosmetics as natural dyes [9]. The naphthoquinone enantiomers alkannin and shikonin have been shown to exhibit strong antioxidant, wound healing, antimicrobial and anti-inflammatory properties, and extensive recent research has adequately established their antitumor properties, indicating the applications of these metabolites as active ingredients in several pharmaceutical and cosmetic preparations. Due to their wide uses they have been efficiently synthesized and have been produced by cell tissue cultures in high yields [9]. In analyses of *Alkanna* species collected from several regions of Greece regarding their constituent hydroxynaphthoquinones and other metabolites, among which was *A. tinctoria*, the main hydroxynaphthoquinones were determined to be β,β-dimethylacrylalkannin, isovalerylalkannin, α-methyl-n-butylalkannin and acetylalkannin [9,10]. Besides naphthoquinones, *A. tinctoria* accumulates PAs, of which 7-angeloylretronecine, triangularine, and dihydroxytriangularine have been previously identified [11]. The antioxidant, sun protection and skin anti-wrinkle effects of different extracts of the root bark of alkanet have been assayed and are widely used based on strong ethnobotanical and ethnopharmacological records from different regions of Greece and Eastern Mediterranean areas [12,13,14,15], confirming it as candidate for cosmetic and pharmaceutical preparations. As a single ingredient or in combination with olive oil and beeswax it was tested in the treatment of burn wounds in a small clinical study [16] and was found to decrease the skin re-epithelialization time. *A. tinctoria* is mostly exploited from the wild, increasing the risk of its extinction, which is why its in vitro propagation has been recently presented [17].

*A. kotschyana* A. DC. (syn *Alkanna amplexicaulis* A. DC.) was first published in A.P. de Candolle, Prodr. 10: 98 (1846). The native range of the species is in South Turkey and is a perennial growing primarily in the subtropical biome [18,19,20]. This species has not been previously researched except in a study on the naphthazarine content of its roots in a comparative study on 16 Turkish *Alkanna* species, where all appeared to contain four known isohexenylnaphthazarin derivatives (namely alkannin, acetylalkannin, deoxyalkannin and β,β-dimethylacrylalkannin), with the latter being the most dominant [21].

In the present study, the methanolic extracts of the aerial parts of the three selected *Alkanna* species are studied for the first time with the aim of the identification of their secondary metabolites and evaluation of their potential biological properties (total flavonoid content, total phenolic content, antioxidant activity and enzyme inhibitory effects).

The correlation between the content of total phenolic and flavonoid compounds and antioxidant activity is well known. Phenolic and flavonoid compounds, functioning as reducing agents and hydrogen donors, possess metal chelating properties that impact antioxidant actions. Furthermore, enzymes are recognized as valuable targets in addressing global health issues due to their involvement in crucial metabolic processes, and discovering such inhibitors derived from plant kingdom holds considerable significance within the scientific community. Acetylcholinesterase and butyrylcholinesterase enzymes play a pivotal role in β-amyloid aggregation, a process linked to Alzheimer’s disease as well as other neurodegenerative diseases, and they are crucial targets in the pursuit of drug discovery for this category of diseases [22]. Additionally in the digestion of carbohydrates, α-amylase and α-glucosidase serve as key enzymes, breaking down starch into oligosaccharides. Inhibiting these enzymes is a significant therapeutic strategy for managing postprandial blood glucose peaks, and medicinal plants play a crucial role in this regard [22]. The phytochemical profiles as well as the bioactivity results of the studied plants indicate that they are promising candidates for further applications.

## 2. Materials and Methods

### 2.1. Plant Material and Methanolic Extract Preparation

The aerial parts of *A. tinctoria*, *A. orientalis* and *A. kotschyana* were collected in Turkey during the summer of 2015. The plants have been identified botanically by Dr. Evren Yildiztugay and voucher specimens have been deposited at the Herbarium of Selcuk University, Turkey. The plant details are provided below. The plant samples were naturally dried in a shaded and well-ventilated environment for approximately 10 days. Following the drying process, the aerial parts of the plant were ground using a laboratory mill, resulting in a particle size of approximately 1 mm. The ground material was then stored at room temperature in dark conditions.

- *Alkanna orientalis* var. *orientalis*: Konya, Hadim-Göksu, 1390 m, Voucher number: GZ-2015-138.
- *Alkanna tinctoria*: Konya, Alaaddin Keykubat Campus-Yükselen village, 1100 m, Voucher number: GZ-2015-118.
- *Alkanna kotschyana*: Adana, Pozantı, Hamidiye village, 1080 m, Voucher number: GZ-2015-125.

### 2.2. Chemicals, Reagents and Equipment

The chemicals were purchased from Sigma-Aldrich (Darmstadt, Germany). They were: 2,2′-azino-bis(3-ethylbenzothiazoline-6-sulphonic acid) (ABTS), 1,1-diphenyl-2-picrylhydrazyl (DPPH), gallic acid, rutin, caffeic acid, electric eel acetylcholinesterase (AChE) (type-VI-S, EC 3.1.1.7), horse serum butyrylcholinesterase (BChE) (EC 3.1.1.8), galantamine, acetylthiocholine iodide (ATChI), butyrylthiocholine chloride (BTChI) 5,5-dithio-bis(2-nitrobenzoic) acid (DTNB), tyrosinase (EC1.14.18.1, mushroom), glucosidase (EC. 3.2.1.20, from *Saccharomyces cerevisiae*), amylase (EC. 3.2.1.1, from porcine pancreas), sodium molybdate, sodium nitrate, sodium carbonate, Folin-Ciocalteu reagent, hydrochloric acid, sodium hydroxide, Trolox, ethylenediaminetetraacetate (EDTA), neocuproine, cupric chloride, ammonium acetate, ferric chloride, 2,4,6-Tris(2-pyridyl)-s-triazine (TPTZ), ammonium molybdate, ferrozine, ferrous sulphate hexahydrate, kojic acid and acarbose. All chemicals were of analytical grade. The spectrophotometric measurements for antioxidant and enzyme inhibition assays were performed using Shimadzu UV-1800 (Shimadzu Corporation, Kyoto, Japan) and Thermo-Multiskan Go (Thermo, USA), respectively. All spectrophotometric measurements were carried out at the room temperature.

### 2.3. Liquid Chromatography/Quadrupole Time-of-Flight Tandem Mass Spectrometry (LC/Q-TOF-MS) Analysis

Phytochemical analyses of the methanolic extracts were performed using LC/Q-TOF-MS system comprising an Agilent 6500 Series Accurate-Mass Quadrupole Time-of-Flight detector (Q-TOF-MS) with ESI-Jet Stream (electrospray ionization method). The Atlantis HILIC silica column (150 × 2.1 mm, dp = 3 μm) from Waters (Milford, MA, USA) was utilized. The chromatographic setup included a diode array detector (DAD), an autosampler, a dual gradient pump and a column heater. The mobile phases consisted of A: H_2_O 1% acetonitrile 0.1% formic acid and B: acetonitrile 95% 0.1% formic acid, both of HPLC grade. The elution gradient was applied at a flow rate of 0.25 mL/min, and the analysis duration was 45 min. The sample injection volume was 10 μL. The experiment employed an electrospray ionization in negative ion mode, and an automated MS/MS system was used for the analysis. Data analysis was performed using Agilent MassHunter software (Version B.08.00, Agilent Technologies, Santa Clara, CA, USA, 2016). Compound identification was accomplished by comparing mass spectra, fragmentation patterns and retention times with those of standards, literature data and available online databases.

### 2.4. Total Phenolic Content (TPC) and Flavonoid Content (TFC)

The total phenolic content was assessed using the Folin–Ciocalteu method, following previously established procedures [22]. A 0.25 mL sample solution was vigorously mixed with 1 mL diluted Folin–Ciocalteu reagent (1:9), and after 3 min, 0.75 mL of 1% Na_2_CO_3_ solution was added. The absorbance of the sample was then measured at 760 nm following a 2 h incubation in the dark at room temperature. The results were expressed in milligrams of gallic acid equivalents per gram of extract (mg GAE/g extract).

The total flavonoid content was assessed using the aluminum trichloride (AlCl_3_) method, following previously established procedures [22]. A 1 mL sample solution was combined with 1 mL of 2% AlCl_3_ in methanol. A blank sample, prepared by adding 1 mL of sample solution to 1 mL of methanol, was also included. After a 10 min incubation at room temperature the absorbances were read at 415 nm. Results were expressed in milligrams of rutin equivalents per gram of extract (mg RE/g extract).

### 2.5. Biological Assays

#### 2.5.1. Radical Scavenging Activity

The methanol extracts were assessed for their ability to scavenge radicals using two methodologies: ABTS^•+^ (2,2′-Azino-bis-(3-ethylbenzothiazoline-6-sulfonic acid) and DPPH^•^ (1,1-diphenyl-2-picrylhydrazyl), following established procedures [22]. Trolox served as a positive control in both assays, and the radical scavenging activity of the examined extracts was quantified as milligrams of Trolox equivalents per gram of extract (mg TE/g extract).

In the ABTS^•+^ assay, the ABTS^•+^ radical cation was generated by incubating a 7 mM ABTS^•+^ solution with 2.45 mM potassium persulfate in darkness at room temperature. The ABTS^•+^ solution was then diluted with methanol to achieve an absorbance of 0.700 ± 0.02 at 734 nm. Subsequently, 1 mL of the sample solution was added to 2 mL of the ABTS^•+^ solution and thoroughly mixed. After 30 min incubation at room temperature, the absorbance of the sample was measured at 734 nm.

In the DPPH^•^ assay, a 1 mL sample solution and 4 mL 0.004% methanol solution of DPPH^•^ were mixed. The absorbance of the sample was then determined at 517 nm following a 30 min incubation in darkness at room temperature.

#### 2.5.2. Reducing Power Assays

The reductive activity of the methanol extracts from the three Alkanna species was determined using the FRAP (Ferric Reducing Antioxidant Power) and CUPRAC (Cupric Ion Reducing Antioxidant Capacity) assays, following the established procedures [22]. The results were quantified in terms of milligrams of Trolox equivalents per gram of extract (mg TE/g extract).

In the FRAP assay, a 0.1 mL sample solution was combined with a 2 mL mixture of acetate buffer (0.3 M, pH 3.6), 2,4,6-tris(2-pyridyl)-s-triazine (TPTZ) (10 mM) in 40 mM HCl, and ferric chloride (20 mM), in a ratio of 10:1:1 (*v*/*v*/*v*). The absorbance of the sample was then read at 593 nm after 30 min incubation at room temperature.

In the CUPRAC assay, a 0.5 mL sample solution was added to a mixture of CuCl_2_ (1 mL, 10 mM), neocuproine (1 mL, 7.5 mM), and NH_4_Ac buffer (1 mL, 1 M, pH 7.0). A blank was prepared using 0.5 mL sample solution with 3 mL of the abovementioned mixture without CuCl_2_. After 30 min incubation at room temperature the absorbance was measured at 450 nm.

#### 2.5.3. Total Antioxidant Capacity and Metal-Chelating Activity

The total antioxidant activity of the samples was assessed using the phosphomolybdenum method, and the results were conveyed in millimoles of Trolox equivalents per gram of extract (mmol TE/g extract). A 0.3 mL sample solution was combined with 3 mL mixture comprising of 0.6 M sulfuric acid, 28 mM sodium phosphate and 4 mM ammonium molybdate. A blank sample of 0.3 mL methanol with 3 mL reagent mixture was also used and the absorbances were recorded at 695 nm following a 90 min incubation at 95 °C.

The metal chelating activity was expressed as milligrams of EDTA equivalents per gram of extract (mg EDTAE/g extract). A 2 mL sample solution was added to 0.05 mL FeCl_2_ solution (2 mM) followed by adding 0.2 mL of 5 mM ferrozine. A 2 mL sample solution with 0.05 mL FeCl_2_ solution and 0.2 mL water, excluding ferrozine, was used as blank sample and the absorbances were measured at 562 nm, after a 10 min incubation at room temperature [22].

#### 2.5.4. Enzyme Inhibitory Activities

The inhibitory effect of methanol extracts from the studied Alkanna species was assessed against cholinesterases (acetylcholinesterase (AChE) and butyrylcholinesterase (BChE)) and it was expressed in milligrams of galantamine equivalents per gram of extract (mg GALAE/g extract). Furthermore, the inhibitory effect of the extracts was assessed against α-amylase and α-glucosidase and it was expressed in millimoles of acarbose equivalents per gram of extract (mmol ACAE/g extract).

Regarding the cholinesterase inhibitory assay, a 50 μL sample solution was combined with 125 μL DTNB and 25 μL AChE or BChE solution in Tris-HCl buffer (pH 8.0). The mixtures were incubated for 15 min at room temperature and the reactions were initiated by adding 25 μL acetylthiocholine iodide or butyrylthiocholine chloride. After 10 min incubation at room temperature, the absorbances were measured at 405 nm.

Regarding the α-amylase inhibitory assay, a 25 μL sample solution was mixed with 50 μL α-amylase solution in phosphate buffer (pH 6.9 with 6 mM sodium chloride) and it was incubated at 37 °C for 10 min before adding 50 mL starch solution (0.05%). The mixture was incubated for 10 min at 37 °C, and the reaction was halted by adding 25 mL HCl (1 M), followed by 100 mL iodine-potassium iodide solution. Similarly, a blank sample without α-amylase was prepared and the absorbances were measured at 630 nm.

In the α-glucosidase inhibitory assay, 50 μL sample solution was mixed with 50 μL glutathione and 50 μL α-glucosidase solution in phosphate buffer (pH 6.8) and 50 μL PNPG. The mixture was incubated for 15 min at 37 °C and the reaction was terminated by adding 50 μL sodium carbonate (0.2 M). A blank sample without α-glucosidase was also prepared in a similar manner and the absorbances were measured at 400 nm [22].

### 2.6. Expression of Results

All analyses were performed in triplicate. The results were expressed as mean values and standard deviation (SD). The differences in the extracts were investigated by using ANOVA (by Tukey) test (*p* < 0.05) and this test was performed in Xlstat 2018.

## 3. Results

### 3.1. Qualitative LC/Q-TOF-MS Analyses

Twenty-eight phenolic metabolites were identified through LC/Q-TOF-MS analysis (Figures S1–S3) of the methanol extracts from the three *Alkanna* species (Table 1). The results obtained from the present study indicated the presence of various caffeic acid derivatives together with flavonoids (flavonols and flavones) and their glycosides.

### 3.2. Biological Evaluation

The total phenolic and flavonoid contents were measured in the methanol extracts of the aerial parts of the three studied *Alkanna* species and the results are presented in Table 2.

Furthermore, their antioxidative capacity was evaluated using different assays, such as DPPH^•^, ABTS^•+^, CUPRAC, FRAP, phosphomolybdenum and a ferrous ion chelating test (Table 2).

Moreover, in the present study, the enzyme inhibitory effects of the Alkanna species were tested against different enzymes, including acetylcholinesterase (AChE), butrylcholinesterase (BChE), tyrosinase, amylase and glucosidase, and the results are summarized in Table 3.

## 4. Discussion

### 4.1. Chemical Analysis of Alkanna Species

The LC/Q-TOF-MS analysis (Table 1) yielded the identification of 28 metabolites among the three studied *Alkanna* species: 2 organic acids, 17 flavonoids (glycosides of quercetin, kaemferol and luteolin) and 9 caffeic acid derivatives (rosmarinic acid, lithospermic acid, salvianolic acids, rabdosiin, and its mono- and di-sodium salts).

*A kotschyana* has not been previously studied for its phenolic and flavonoid content except for a study on the naphthazarine content of its roots [21].

The identified metabolites in the aerial parts of both *A. orientalis* and *A. tinctoria* have been compared with existing data from the literature [3,4,5,31] (Table 4). It has been observed that one organic acid, eight phenolic compounds and six flavonoids are reported for the first time in the aerial parts of these species in the current study. It is noteworthy that although caffeic acid has been referred to once in the genus [31] previously, the caffeic acid derivatives (rosmarinic acid, lithospermic acid, salvianolic acids and rabdosiin and its salts) are completely missing in the studied species.

Among the identified organic acids which are widely distributed in the plant kingdom, only malic acid has been referred to before [31]. Caffeic acid, along with quercetin and kaempferol glycosides, has been identified in various Boraginaceae species previously [32].

Rosmarinic acid, a caffeic acid derivative, is commonly found in plants of the Boraginaceae family, and could serve as a chemotaxonomic marker [1]. It is well known for its extensive range of bioactivities, including antioxidant, antiviral, antimicrobial and anti-inflammatory properties. Notably, it primarily exhibits neuroprotective, anti-acetylcholinesterase and hepatoprotective properties [32].

Additionally, this study includes the annotation of rabdosiin, a dimer of rosmarinic acid, and its disodium salt, which is a novel compound recently isolated and structurally determined by our team from *Alkanna sfikasiana* [1]. Moreover, salvianolic acids, which are caffeic acid derivatives, proposed to be biosynthetically related to rosmarinic acid and usually reported in Boraginaceae plants [33], are also identified.

The detection of several methylated derivatives of kaempferol and quercetin [kaempferol-3,4′-dimethylether (ermanin), kaempferol-3,7-dimethylether (kumatakenin), 6-methoxyquercetin-3,3′-dimethylether (jaceidin), 6-methoxykaempferol-3-methyl ether, 6-methoxykaempferol-3,7-dimethylether (penduletin) and kaempferol-3-methylether (isokaempferide)]) is in accordance with the previous literature on *A. orientalis* from Saudi Arabia [5].

Furthermore, derivatives of kaempferol and quercetin (kaempferol-3-glucoside, kaempferol-3-rutinoside, kaempferol-3,6-dimethyl ether and its 7-glucoside, quercetin-3-glucoside, quercetin-3-rutinoside) have previously been isolated in *A. orientalis* from the Sinai peninsula, Egypt [4]. According to the comparison (Table 4) it is clear that luteolin derivatives have not been referred to so far in the literature, and have been reported only in the current study.

Caffeic, chlorogenic and rosmarinic acids have also been detected in the roots of Boraginaceae family members [27,34], and before now caffeic acid has only been identified previously in *A. tinctoria* from Bulgaria. The absence of such metabolites which mainly characterize Boraginaceae plants could be explained as a result of studies focused only on flavonoid content rather than the determination of rosmarinic acids’ existence. Moreover, the presence of these secondary metabolites may vary across different plant organs due to differences in biosynthetic pathways.

### 4.2. Biological Evaluation

#### 4.2.1. Total Phenolic and Flavonoid Content

The total phenolic and flavonoid contents were measured in the methanol extracts of the studied species and the highest total phenolic content was detected in the methanol extract of *A. tinctoria* (53.36 mg GAE/g), followed by *A. kotschyana* (43.93 mg GAE/g) and *A. orientalis* (34.92 mg GAE/g). However, *A. orientalis* was the richest in terms of total flavonoid content with 25.90 mg RE/g. Different values for the total bioactive compounds in the members of the genus *Alkanna* have been reported in the literature [8,35,36]. These differences can be explained by agroclimatic conditions such as rainfall, altitude and soil composition, as well as extraction solvents used.

#### 4.2.2. Antioxidant Activity

Natural extracts with antioxidant properties, especially those derived from plants, are in great demand in the market due to the harmful effects of synthetic compounds on human health [37]. The antioxidative capacity of the methanol extracts from the aerial parts of three *Alkanna* species was evaluated using different assays, such as DPPH^•^, ABTS^•+^, CUPRAC, FRAP, phosphomolybdenum and ferrous ion chelating test.

DPPH and ABTS are used to measure the ability of the tested extracts to scavenge radicals and to determine whether the extracts donate a hydrogen to radicals and thus quench them. As shown in Table 2, the best radical scavenging ability was recorded in *A. tinctoria* (DPPH: 211.58 mg TE/g; ABTS: 366.88 mg TE/g), followed by *A. kotschyana* and *A. orientalis.* The ability of antioxidants to donate electrons is evaluated by researchers using reducing power assays, such as CUPRAC and FRAP. Similar to radical scavenging assays, the strongest ability was found in *A. tinctoria* with 384.78 mg TE/g and 290.51 mg TE/g by CUPRAC and FRAP assays, respectively. The observed superior ability of *A. tinctoria* can be attributed to a higher concentration of phenolics in the extract. Furthermore, according to Table 1, salvianolic acid A was only detected in *A. tinctoria* and the compound may contribute to the observed antioxidant properties. Consistent with our results, these compounds have been reported as significant antioxidants by several researchers [38,39,40]. However, the total antioxidant ability in the phosphomolybdenum assay can be ranked as *A. orientalis* > *A. tinctoria* > *A. kotschyana*. The ability to chelate metals is associated with preventing the production of hydroxyl radicals in the Fenton reaction, and this is also known to be an important antioxidant mechanism. *A. orientalis* (19.11 mg EDTAE/g) had the best metal chelation ability, followed by *A. tinctoria* and *A. kotschyana*. The order obtained from phosphomolybdenum and metal chelating assays were different from the radical scavenging and reducing power assays.

#### 4.2.3. Enzyme Inhibitory Activity

Enzyme inhibition is gaining growing significance in pharmaceutical applications to alleviate the symptoms of serious health problems such as diabetes, obesity and Alzheimer’s disease. In this sense, the discovery of novel enzyme inhibitors is a crucial topic in scientific studies [41]. In the present study, the enzyme inhibitory effects of the *Alkanna* species were tested against different enzymes including acetylcholinesterase (AChE), butrylcholinesterase (BChE), tyrosinase, amylase and glucosidase. Inhibition of AChE and BChE is associated with an increase in acetylcholine levels in synaptic gap and could help improve cognitive function in Alzheimer’s patients. Table 3 shows that *A. tinctoria* and *A. kotschyana* showed inhibition against both enzymes. However, *A. orientalis* was not active in these enzymes. Caffeic acid has been reported to be a significant inhibitor of both of these enzymes [42,43]. Amylase and glucosidase are the main enzymes in the hydrolysis of carbohydrates and their inhibition can control blood sugar levels in diabetics. All *Alkanna* species inhibited these enzymes and the best inhibitory effect was achieved by *A. orientalis*. The observed abilities can be attributed to the presence of some compounds. For example, rosmarinic acid showed greater inhibition of these enzymes in in vivo and in vitro studies [44,45]. In addition, quercetin and its glucoside are known as natural antidiabetic agents [46,47,48]. Taken together, the tested *Alkanna* species can be considered as potential raw materials for natural sources of enzyme inhibitors.

## 5. Conclusions

The aerial parts of three *Alkanna* species growing wild in the South East Mediterranean basin in Turkey (*A. orientalis*, *A. tinctoria* and *A. kotschyana*) were analyzed by LC-Q-TOF-MS, leading to the identification of 28 secondary metabolites, most of them common to the three studied species, among which 15 were described for the first time. The analysis revealed a complex metabolite composition of the investigated species, showcasing numerous phenolic acids and flavonoids. The biological properties of the investigated species have been thoroughly assessed, including the in vitro exploration of their antioxidant, neuroprotective and antidiabetic potential by different methods (TPC, TFC, DPPH, ABTS, CUPRAC, FRAP, phosphomolybdenum and ferrous ion chelating test, AChE, BChE, α-amylase and α-glucosidase).

*A. kotschyana* was studied phytochemically for the first time as it has only recently been identified botanically. *A. tinctoria* showed the highest antioxidant activity whereas *A. orientalis* exhibited the most interesting inhibitory capacity, especially against α-amylase and α-glucosidase enzymes. Several phenolic metabolites such as luteolin type flavonoids as well as caffeic acid derivatives were reported for the first time.

In addition to the significance of the phytochemical analysis in the field of chemotaxonomy, this research makes substantial contributions to understanding the chemical and multi-biological characteristics of the studied aerial parts of *Alkanna* species, as until now most of the existing research works have been focused on the roots of the plants due to their content in highly commercially important naphthoquinone-type metabolites (shikonins and alkannins). Consequently, these plants emerge as promising sources of bioactive metabolites, presenting valuable prospects for further exploration in the realms of phytotherapeutic research or application within the field of cosmetology. This potential comes from the wide acceptance of several *Alkanna*–based final products mostly for external/dermatological uses, provided that appropriate safety tests are conducted.

## Figures and Tables

**Table 1:** LC/Q-TOF-MS analyses of 3 *Alkanna* species’ methanol extracts.

Identified Metabolites		Molecular Formula	[M − H]	*Alkanna orientalis*	*Alkanna tinctoria*	*Alkanna kotschyana*	Ref.
Gluconic acid	organic acid	C_6_H_12_O_7_	195.0505	*√*	*√*	*√*	[23]
Malic acid	organic acid	C_4_H6O_5_	133.0139	*√*	*√*	*√*	[24]
Caffeic acid	phenolic acid	C_9_H_8_O_4_	179.0349	*√*	*√*	*√*	[25]
Lithospermic acid	phenolic acid	C_27_H_22_O_12_	537.1051	*√*	*√*	-	[23]
Quercetin-3-rutinoside (rutin)	flavonol	C_27_H_30_O_16_	609.1409	*√*	*√*	*√*	[24]
Luteolin-7-glucopyranoside	flavone	C_21_H_20_O_11_	447.093	*√*	*√*	*√*	[26]
Quercetin-3-glucopyranoside	flavonol	C_21_H_20_O_12_	463.0854	*√*	*√*	*√*	[27]
Quercetin-3-galactopyranoside	flavonol	C_21_H_20_O_12_	463.0854	*√*	*√*	*√*	[28]
Luteolin-7-rutinoside	flavone	C_15_H_10_O_6_	593.1484	*√*	*√*	*√*	[26]
Kaempferol-3-rutinoside	flavonol	C_27_H_30_O_15_	593.1474	*√*	*√*	*√*	[29]
Kaempferol-3-glucopyranoside (astragalin)	flavonol	C_21_H_20_O_11_	447.0897	*√*	*√*	*√*	[29]
Quercetin-3-acetyl-glucopyranoside	flavonol	C_23_H_22_O_13_	505.0995	*√*	*√*	*√*	[30]
Rabdosiin	phenolic compound	C_36_H_30_O_16_	717.1408	*√*	*√*	*√*	[1]
Monosodium salt of rabdosiin	phenolic compound	C_36_H_29_O_16_Na	739.1225	*√*	*√*	*√*	[1]
Disodium salt of rabdosiin	phenolic compound	C_36_H_30_O_16_Na_2_	741.1407	*√*	*√*	*√*	[1]
Rosmarinic acid	phenolic acid	C_18_H_16_O_8_	359.0795	*√*	*√*	*√*	[23]
5,7,4′-trihydroxy-3,6,8-trimethoxyflavone (sarothrin)	flavone	C_18_H_16_O_8_	359.0783	*√*	-	-	[3]
Lithospermic acid B/Salvianolic acid B	phenolic acid	C_36_H_30_O_16_	717.1414	*√*	-	-	[25]
Kaempferol-3,4′-dimethylether (ermanin)	flavonol	C_17_H_14_O_6_	313.0571	*√*	*√*	*√*	[5]
Kaempferol-3,7-dimethylether (kumatakenin)	flavonol	C_17_H_14_O_6_	313.0571	*√*	*√*	*√*	[5]
Kaempferol-3-acetyl-glucopyranoside	flavonol	C_23_H_22_O_12_	489.1020	*√*	*√*	*√*	[28]
Salvianolic acid C	phenolic acid	C_26_H_20_O_10_	491.0940	*√*	*√*	-	[25]
Luteolin	flanone	C_15_H_10_O_6_	285.0407	*√*	*√*	*√*	[26]
Kaempferol-3-methylether (isokaempferide)	flavonol	C_16_H_12_O_6_	299.0559	*√*	*√*	*√*	[5]
6-methoxyquercetin-3,3′-dimethylether (jaceidin)	flavonol	C_18_H_16_O_8_	359.0748	*√*	*√*	*√*	[5]
6-methoxykaempferol -3-methylether	flavonol	C_17_H_14_O_7_	329.0545	*√*	*√*	*√*	[5]
6-methoxykaempferol-3,7-dimethylether (penduletin)	flavonol	C_18_H_16_O_7_	343.0805	*√*	*√*	*√*	[5]
Salvianolic acid A	phenolic acid	C_26_H_22_O_10_	493.1107	-	*√*	-	[25]

*√*: Present; -: Absent.

**Table 2:** Antioxidant properties of methanol extracts from three studied *Alkanna* species *.

Assays	*Alkanna orientalis*	*Alkanna tinctoria*	*Alkanna kotschyana*
Total bioactive components			
Total phenolic content (mg GAE/g extract)	34.92 ± 0.85 ^c^	53.36 ± 0.80 ^a^	43.93 ± 1.01 ^b^
Total flavonoid content (mg RE/g extract)	25.90 ± 0.83 ^a^	20.79 ± 0.58 ^b^	15.97 ± 0.46 ^c^
Antioxidant assays			
DPPH^•^ (mg TE/g extract)	82.98 ± 1.40 ^c^	211.58 ± 2.63 ^a^	131.02 ± 3.14 ^b^
ABTS^•+^ (mg TE/g extract)	192.41 ± 4.15 ^c^	366.88 ± 3.25 ^a^	250.61 ± 4.01 ^b^
FRAP (mg TE/g extract)	92.13 ± 2.23 ^c^	290.51 ± 9.21 ^a^	261.05 ± 3.26 ^b^
CUPRAC (mg TE/g extract)	160.12 ± 2.89 ^c^	384.78 ± 5.55 ^a^	371.65 ± 6.19 ^b^
Phosphomolybdenum (mmol TE/g extract)	1.70 ± 0.01 ^a^	1.61 ± 0.15 ^a^	1.51 ± 0.03 ^b^
Metal chelating (mg EDTAE/g extract)	19.11 ± 0.08 ^a^	6.70 ± 0.28 ^b^	4.86 ± 0.22 ^c^

* Values are reported as mean ± SD of three parallel experiments. GAE: Gallic acid equivalent; RE: Rutin equivalent; TE: Trolox equivalent; EDTAE: EDTA equivalent. Different superscripts ^(a–c)^ indicate significant differences in the extracts (*p* < 0.05).

**Table 3:** Enzyme inhibitory activity of methanol extracts from three studied *Alkanna* species.

Assays	*Alkanna orientalis*	*Alkanna tinctoria*	*Alkanna kotschyana*
AChE inhibition (mg GALAE/g extract)	na	1.51 ± 0.01 ^b^	1.67 ± 0.06 ^a^
ΒChE inhibition (mg GALAE/g extract)	na	1.29 ± 0.08 ^a^	1.09 ± 0.11 ^b^
α-amylase inhibition (mmol ACAE/g extract)	0.61 ± 0.06 ^a^	0.45 ± 0.01 ^c^	0.49 ± 0.01 ^b^
α-glucosidase inhibition (mmol ACAE/g extract)	6.49 ± 0.16 ^a^	4.43 ± 0.27 ^b^	3.69 ± 0.13 ^c^

Values are reported as mean ± SD of three parallel experiments. GALAE: Galanthamine equivalent; ACAE: Acarbose equivalent. Different superscripts ^(a–c)^ indicate significant differences in the extracts (*p* < 0.05); na = not active.

**Table 4:** Comparison of chemical profile (organic acids, phenolic compounds, flavonoids) of the studied species with existing data from the literature.

	Identified Metabolites	*A. orientalis* Turkey	*A. orientalis* Cultivated [3]	*A. orientalis* Egypt [4]	*A. orientalis* Saudi Arabia [5]	*A. tinctoria* Turkey	*A. tinctoria* Bulgaria [31]
Organic acids	Gluconic acid	*√*				*√*	
	Malic acid	*√*				*√*	*√*
	Succinic acid						*√*
	Glyceric acid						*√*
Phenolic compounds	Caffeic acid	*√*				*√*	*√*
	4-Hydroxybenzoic acid						*√*
	Vanilic acid						*√*
	cis 4-Hydroxycinnamic acid						*√*
	Protocatechuic acid						*√*
	trans 4-Hydroxycinnamic acid						*√*
	Ferulic acid						*√*
	Rosmarinic acid	*√*				*√*	
	Lithospermic acid	*√*				*√*	
	Lithospermic acid B/Salvianolic acid B	*√*					
	Salvianolic acid C	*√*				*√*	
	Salvianolic acid A					*√*	
	Rabdosiin	*√*				*√*	
	Monosodium salt of rabdosiin	*√*				*√*	
	Disodium salt of rabdosiin	*√*				*√*	
Flavonoids	Quercetin-3-rutinoside (rutin)	*√*		*√*		*√*	
	Quercetin-3-glucopyranoside	*√*		*√*		*√*	
	Quercetin-3-galactopyranoside	*√*				*√*	
	Quercetin-3-acetyl-glucopyranoside	*√*				*√*	
	Luteolin	*√*				*√*	
	Luteolin-7-rutinoside	*√*				*√*	
	Luteolin-7-glucopyranoside	*√*				*√*	
	Kaempferol-3-rutinoside	*√*		*√*		*√*	
	Kaempferol-3-glucopyranoside (astragalin)	*√*		*√*		*√*	
	Kaempferol-3,4′-dimethylether (ermanin)	*√*			*√*	*√*	*√*
	Kaempferol-3,7-dimethylether (kumatakenin)	*√*			*√*	*√*	*√*
	Kaempferol-3-acetyl-glucopyranoside	*√*				*√*	
	Kaempferol-3-methylether (isokaempferide)	*√*			*√*	*√*	*√*
	6-methoxyquercetin-3,3′-dimethylether (jaceidin)	*√*				*√*	*√*
	6-methoxykaempferol -3-methylether	*√*		*√*		*√*	
	6-methoxykaempferol -3-methylether-glucoside			*√*			
	6-methoxykaempferol-3,7-dimethylether (penduletin)	*√*			*√*	*√*	*√*
	5,7,4′-trihydroxy-3,6,8-trimethoxyflavone (sarothrin)	*√*	*√*				

*√*: Present.

## Data Availability

Data are contained within the article and Supplementary Materials.

## Supplementary Materials

The following are available online at https://www.mdpi.com/article/10.3390/plants13020278/s1, Figure S1. LC-MS chromatogram of *Alkanna orientalis*; Figure S2. LC-MS chromatogram of *Alkanna tinctoria*; Figure S3. LC-MS chromatogram of *Alkanna kotschyana*.

## Abbreviations

AChE	Acetylcholinesterase
BChE	Butyrylcholinesterase
DPPH	2,2-Diphenyl-1-Picrylhydrazyl
ABTS	2,2′-Azino-bis-3-ethylbenzothiazoline-6-sulfonic acid
TE	Trolox Equivalents
RE	Rutin Equivalents
GAE	Gallic Acid Equivalents
LC/Q-TOF-MS	Liquid Chromatography/Quadrupole Time-of-Flight Tandem Mass Spectrometry
CUPRAC	Cupric Ion Reducing Antioxidant Capacity
FRAP	Ferric Reducing Antioxidant Power
GC	Gas Chromatography
GC-MS	Gas Chromatography–Mass Spectrometry
MIC	Minimum Inhibitory Concentration
TPC	Total Phenolic Content
TFC	Total Flavonoid Content
GALAE	Galantamine Equivalents
ACAE	Acarbose Equivalents
EDTAE	EDTA Equivalents
