# Supplementary material for: Phytochemical Profiling and Biological Assessment of the Aerial Parts from Three Mediterranean Alkanna Species (A. orientalis, A. tinctoria, A. kotschyana) in the Boraginaceae Family
Source: Plants (Basel). 2024 Jan 17;13(2):278. doi: 10.3390/plants13020278 (PMC10818510; doi:10.3390/plants13020278)
Supplement: Supplementary file 1 [file plants-13-00278-s001.zip › plants-2758489-supplementary.pdf]

plants

Supplementary File

Phytochemical Profiling and Biological Assessment of the Aerial Parts from Three Mediterranean *Alkanna* Species (*A. orientalis*, *A. tinctoria*, *A. kotschyana*) in the Boraginaceae Family

Christos Ganos [1], Gökhan Zengin [2], Ioanna Chinou [1], Nektarios Aligiannis [1] and Konstantia Graikou [1,*]

[1] Laboratory of Pharmacognosy and Chemistry of Natural Products, Department of Pharmacy, National & Kapodistrian University of Athens, Zografou, 15771 Athens, Greece; chris50ganos@hotmail.com (C.G.); ichinou@pharm.uoa.gr (I.C.); aligiannis@pharm.uoa.gr (N.A.)
[2] Laboratory of Physiology and Biochemistry, Department of Biology, Science Faculty, Selcuk University, 42130 Konya, Turkey; gokhanzengin@selcuk.edu.tr
* Correspondence: kgraikou@pharm.uoa.gr

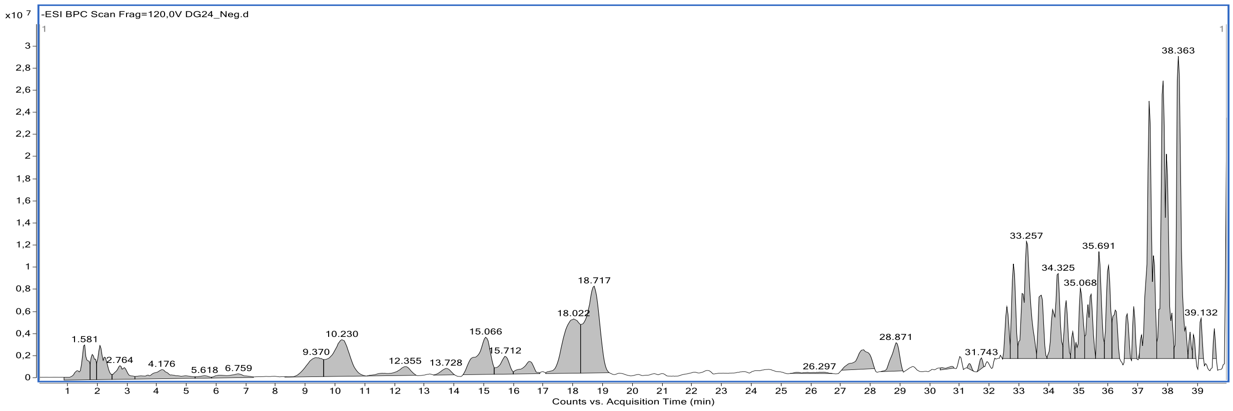

Figure S1. LC-MS chromatogram of *Alkanna orientalis*.

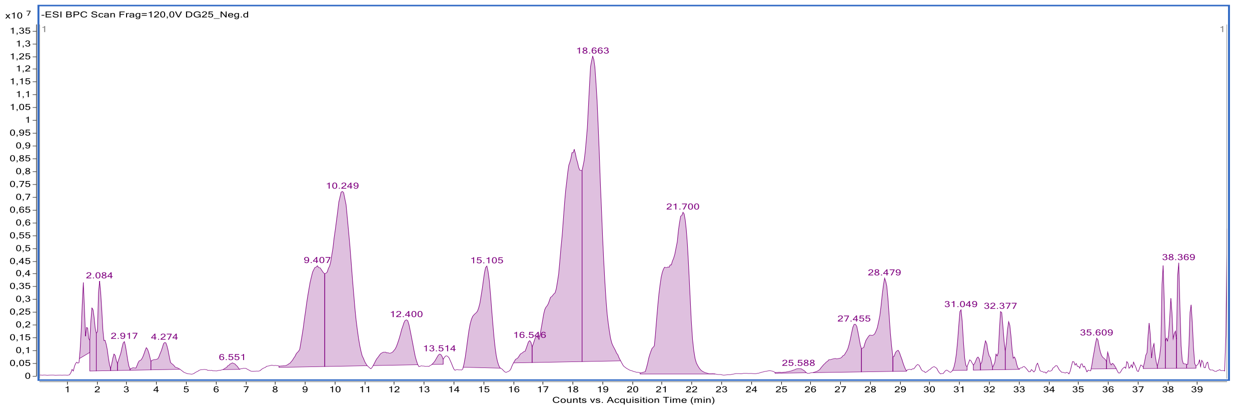

Figure S2. LC-MS chromatogram of *Alkanna tinctoria*.

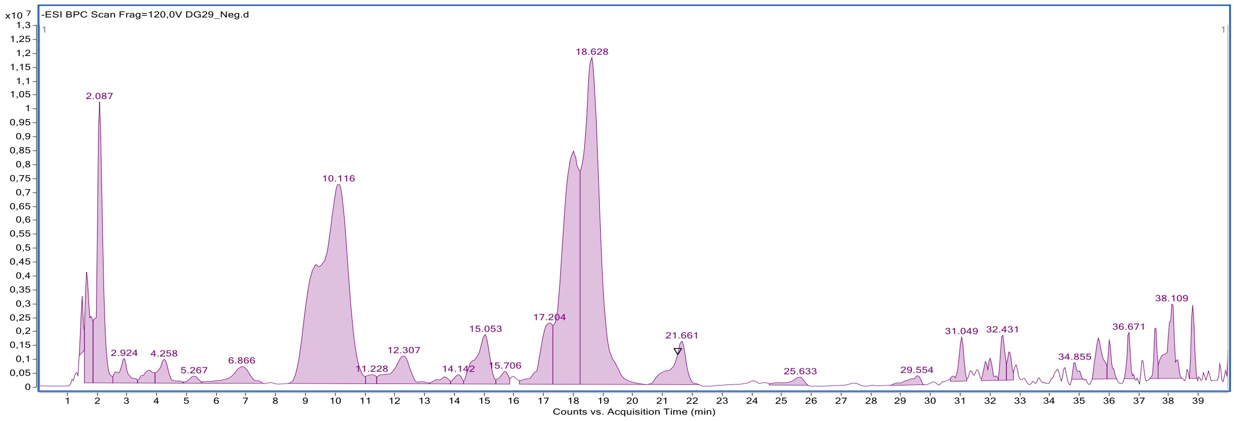

Figure S3. LC-MS chromatogram of *Alkanna kotschyana*.
